# Effects of stale maize on growth performance, immunity, intestinal morphology and antioxidant capacity in broilers

**DOI:** 10.5713/ajas.19.0224

**Published:** 2019-08-03

**Authors:** J. B. Liu, H. L. Yan, Y. Zhang, Y. D. Hu, H. F. Zhang

**Affiliations:** 1 School of Life Science and Engineering, Southwest University of Science and Technology, Mianyang, Sichuan 621010, China; 2 State Key Laboratory of Animal Nutrition, Institute of Animal Sciences, Chinese Academy of Agricultural Sciences, Beijing 100193, China; 3 Farm Animal Genetic Resources Exploration and Innovation Key Laboratory of Sichuan Province, Sichuan Agricultural University, Ya’an, Sichuan 625014, China

**Keywords:** Antioxidant Function, Broilers, Immunity, Stale Maize

## Abstract

**Objective:**

This study was conducted to determine the effects of stale maize on growth performance, immunity, intestinal morphology, and antioxidant capacity in broilers.

**Methods:**

A total of 800 one-day-old male Arbor Acres broilers (45.4±0.5 g) were blocked based on body weight, and then allocated randomly to 2 treatments with 20 cages per treatment and 20 broilers per cage in this 6-week experiment. Dietary treatments included a basal diet and diets with 100% of control maize replaced by stale maize.

**Results:**

The content of fat acidity value was higher (p<0.05) while the starch, activities of catalase and peroxidase were lower (p<0.05) than the control maize. Feeding stale maize diets reduced (p<0.05) average daily feed intake (ADFI) throughout the experiment, feed conversion ratio (FCR) during d 0 to 21 and the whole experiment as well as relative weight of liver, spleen, bursa of Fabricius and thymus (p<0.05) on d 21. Feeding stale maize diets decreased jejunum villus height (VH) and VH/crypt depth (CD) (p<0.05) on d 21 and 42 as well as ileum VH/CD on d 42. The levels of immunoglobulin G, acid α-naphthylacetate esterase positive ratios and lymphocyte proliferation on d 21 and 42 as well as lysozyme activity and avian influenza antibody H_5_N_1_ titer on d 21 decreased (p<0.05) by the stale maize. Feeding stale maize diets reduced (p<0.05) serum interferon-γ, tumor necrosis factor-α, interleukin-2 on d 21 and interleukin-6 on d 21 and 42. Broilers fed stale maize diets had lower levels of (p<0.05) total antioxidative capacity on d 42, superoxide dismutase and glutathione peroxidase on d 21 and 42, but higher (p<0.05) levels of malondialdehyde on d 21 and 42.

**Conclusion:**

Feeding 100% stale maize decreased ADFI and FCR, caused adverse effects on immunity and antioxidant function and altered intestinal morphology in broilers.

## INTRODUCTION

Grains are the main source of energy in diets used for poultry due to high energy content and digestibility as well as low anti-nutritional factors [1]. A large amount of grain is stored to cope with food shortages caused by various natural disasters according to the temporary purchasing and storage policy in China. Maize is an indispensable reserve grain. It is estimated that presently there are more than 200 million tons stale maize, which might be stored for more than 3 or 4 years in China. This stale maize may be eventually used in livestock feed, food processing and industrial ethanol production [2–4].

Due to its high content of polyunsaturated fatty acid (PUFA), maize is prone to oxidative rancidity during storage [5]. Several studies have demonstrated that after long-term storage, the activities of catalase (CAT) and peroxidase (POD) as well as the content of oleic acid and linoleic acid in maize decreased, while the levels of free fatty acids (FFAs) increased [6–8]. The oxidized stale maize and its peroxide products can lead to oxidative stress, which may cause excessive oxidation of the body tissues and produce highly reactive molecules such as reactive oxygen radicals and reactive nitrogen species [8–9]. The oxidative stress caused by excessive free radicals involves a series of synthetic reactions including physiology, biochemistry and immunity, which may depress the performance and health of livestock [10]. Besides, the stored maize may cause starch degradation and lead to the formation of resistant starch [11]. In the process of maize storage, the mycotoxin content may increase in addition to the changes in its own physical and chemical properties, which may further affect its nutritive value and restrict the application of stale maize [12].

Several studies have evaluated the effect of storage duration on the nutritional value of maize from the utilization of their energy or proteins [13–15]. Previous study reported no loss in feeding value in rats fed stale maize stored up to 6 years [13]. The apparent metabolizable energy corrected for nitrogen and chemical composition of stale maize stored for 9 years under proper conditions did not change in broilers at 15 to 17 d of age [15]. The data obtained above may indicate that storage under adequate conditions does not alter the nutritional value of maize. On the contrary, the length of storage had an adverse effect on energy content due to the decreased fat content and the increased FFAs [14].

To the best of our knowledge, little information on broilers fed stale maize diets is available. Broilers may be particularly susceptible to oxidative stress due to the genetic selection toward lean and large breast muscles and fast growth rates [16]. Therefore, the objective of this study was to investigate the influence of feeding stale maize on growth performance, relative organ weight, immune function, intestinal morphology and antioxidant capacity in broilers.

## MATERIALS AND METHODS

### Analysis of maize

All maize samples with the same hybrid type used in the current study were purchased from a typical national brick barn, each of which could hold up to 5,000 tons of maize (Mianyang, Sichuan, China). The control maize and stale maize samples stored at stable conditions according to the national regulatory rules for 1 and 4 years after harvest, respectively. The moisture, unit weight, starch, crude protein (CP) and fat acidity value were determined according to AOAC [17]. The activities of CAT and POD were measured according to previous study [18]. The fatty acid content was analyzed by gas chromatography with a flame ionization detector (Hewlett Packard 5890 Series II, Palo Alto, CA, USA) according to the method described previously [19]. The mycotoxins were measured by enzyme-linked immunosorbent assay (ELISA) method (kits, Neogen Company, MN, USA; MicroplateReader, Model 680, Bio-Rad, Hercules, CA, USA) according to the manufacturer’s instructions.

### Experimental design and broiler husbandry

The Animal Welfare Committee of Southwest University of Science and Technology approved the animal care protocol used for this experiment (SWUST20180154). A total of 800 one-day-old male Arbor Acres broilers with an average initial body weight (BW) of 45.4±0.5 g were blocked on the basis of BW and placed in stainless steel battery brooders (2.0×1.55 m). All broilers were housed in an environmentally controlled facility. The environmental temperature began at 33°C for the first 3 d and was then reduced to 24°C until the end of the experiment and the relative humidity was around 60%.

This 6-wks experiment consisted of 2 treatments with 20 cages per treatment and 20 broilers per cage in a randomized complete block design. A 2-phase feeding program was used: a starter diet from d 1 to 21 and a grower diet from d 22 to 42. All diets (Table 1) were formulated to meet or exceed the NRC requirements for broilers [20], and the dietary treatments were: control (CON) and 100% stale maize. Diets were fed in pellet form and feed and water were provided *ad libitum* throughout the experiment.

Proximate analysis of diets for dry matter (DM) and ash was carried out according to AOAC [17]. The DM of the feed was determined after drying for 24 h at 105°C. Ash was determined after ignition of a weighed sample in a muffle furnace (Nabertherm, Bremen, Germany) at 550°C for 6 h. The ash was then digested in *aqua regia* (HCl/HNO_3_ mixture), and the solution was used for phosphorus (P) and calcium (Ca) determination. Ca concentration was determined using an atomic absorption spectrophotometer (Varian’50, Varian, Palo Alto, CA, USA), and the concentration of P was determined spectrophotometrically (NanoDrop 2000c, Thermo Scientific, Waltham, MA, USA) with some modification [21].

### Sampling and measurements

The broilers were weighed, and feed intake was recorded on d 1, 21, and 42, and average daily gain (ADG), average daily feed intake (ADFI), and feed conversion ratio (FCR) were calculated.

At the end of the third week and the experiment, 4 broilers were randomly selected from each cage and blood samples were collected from the jugular vein into a sterile syringe and stored at −4°C. Blood samples were then centrifuged at 3,000×g for 15 min and serum was separated. The levels of superoxide dismutase (SOD), total antioxidative capacity (T-AOC), malondialdehyde (MDA), glutathione peroxidase (GSH-PX), immunoglobulin G (IgG), interleukin 2 (IL-2), IL-6, tumor necrosis factor-α (TNF-α) and interferon-γ (IFN-γ) in the serum were analyzed using ELISA method (Jiancheng Biotechnology Institute, Nanjing, China) described previously [22]. The peripheral blood T lymphocyte proliferation and acid α-naphthylacetate esterase positive ratios (ANAE+) of the peripheral blood T lymphocytes was determined according to previous study [23]. Lysozyme activity was measured by the reduction speed of its substrate (*Micrococcus lysodeikticus*), which was reflected by the change in transmissivity at the wavelength of 530 nm following the manufacturer’s recommendation (Jiancheng Biotechnology Institute, China). The specific antibody titer of broilers (avian influenza H_5_N_1_ antibody) was determined by HA/HI method [24]. After blood collection, the same broilers were weighed individually, and then sacrificed by cervical dislocation. The liver, proventriculus, gizzard, pancreas, thymus, bursa of Fabricius, spleen, duodenum, jejunum and ileum were removed by trained personnel and weighed after flushing with saline. Organ size was expressed as a percentage of BW.

After weighing, the samples of small intestine tissues (ap proximately 2 cm from duodenum, jejunum, and ileum, respectively) were collected for determination of mucosal morphology. The tissues from duodenum, jejunum, and ileum, respectively, were cleaned with saline and then fixed in 10% neutral formalin. The fixed tissues were trimmed, embedded in paraffin for mucosal morphology and integrity. Thin sections (5 μm) were sliced and mounted on slide, and then stained with haematoxylin and eosin (H&E staining procedure) for histopathological examination by an optical microscope (Olympus, Tokyo, Japan) using the method described previously [25]. Intestinal morphological measurements included the following 3 indices: villus height (VH), crypt depth (CD), and VH/CD. These indexes were quantified according to the method described previously [26]. Mean values of VH, CD, and VH/CD within each segment (eight villi per bird) were calculated.

### Statistical analysis

Data were analyzed by analysis of variance using the T-test procedure of SAS (SAS Inst. Inc., Cary, NC, USA) with the cage being the experimental unit. Variability in the data is expressed as the standard error of means. Probability values less than 0.05 were considered significant.

## RESULTS

### Nutrient content and trait of maize

There were no differences (p>0.05) in the moisture, unit weight, content of CP and linolenic acid in stale maize compared with the control maize (Table 2). The starch, CAT and POD activities, oleic acid and linoleic acid reduced (p<0.05) while fat acidity increased (p<0.05) in stale maize compared with the control maize. All the detected mycotoxins did not differ (p>0.05) or exceed the regulatory guidance concentration of Chinese National Standard (GB 13078-2001; GB 13078.2-2006), European Commission and FAO [27,28] between the control and stale maize.

### Growth performance

During d 0 to 21, feeding stale maize diets reduced ADFI and FCR (p<0.05), while did not affect ADG (p>0.05; Table 3). During d 22 to 42, ADFI was decreased (p<0.05) by the stale maize treatment, while ADG and FCR did not differ (p>0.05). In the whole experiment, broilers fed stale maize diets had lower ADFI and FCR (p<0.05), but the dietary treatment did not affect the final BW and ADG (p>0.05).

### Relative organ weight

On d 21, the relative weight of liver, spleen, bursa of Fabricius and thymus in broilers fed stale maize diets decreased (p< 0.05; Table 4). On d 42, feeding stale maize diets increased (p<0.05) the relative weight of pancreas and gizzard, whereas the relative weight of spleen decreased in the stale maize treatment (p<0.05).

### Intestinal mucosal morphology

The dietary treatment did not influence (p>0.05) VH, CD, or VH/CD in duodenum on d 21 or 42 (Table 5). In jejunum, the VH and VH/CD was decreased by the stale maize treatment on d 21 and 42 (p<0.05). The VH/CD in ileum was reduced in broilers fed stale maize diets (p<0.05) on d 42, whereas others did not differ (p>0.05) on d 21 or 42.

### Serum immunological parameters

Feeding stale maize diets decreased (p<0.05) IgG, ADAE+, and lymphocyte proliferation on d 21 and 42 as well as lysozyme activity and avian influenza antibody H_5_N_1_ titer on d 21 (Table 6).

### Serum cytokines

Broilers fed stale maize diets had lower (p<0.05) levels of serum IFN-γ, TNF-α, IL-2 on d 21 and IL-6 on d 21 and 42 (Table 7).

### Serum antioxidant capacity

Feeding stale maize diets decreased (p<0.05) T-AOC on d 42, SOD and GSH-PX on d 21 and 42 (Table 8), whereas the content of MDA in the stale maize treatment increased (p< 0.05) on d 21 and 42.

## DISCUSSION

### Nutrient content and trait of maize

As expected, in the current study, the starch, CAT and POD activities, oleic acid and linoleic acid in stale maize decreased, which agreed with previous results [29]. Similar findings were observed by other researchers [5,9]. The stale maize decreased CAT and POD activities and presented time-dependent effect [29]. Previous findings suggest the maximum period for maize storage should be 4 years [30]. The CAT and POD activities can be used as indexes for evaluating the quality of stored maize [9]. This was the main reason for the choice of the stale maize stored for 4 years in the present study. Maize can be suitable for storage when the POD activity is higher than 400 U/g per min, while maize with a value between 250 and 400 U/g per min and below 250 U/g per min may be not suitable for storage [9]. In agreement with previous studies, we found that the POD value in the maize stored for 1 and 4 years was 510 and 191 U/g per min, respectively, in the current study. Furthermore, the fat acidity increased and fatty acids (C18:1 and C18:2) decreased in the stale maize in our study. In agreement with our results, previous studies found that with the degradation of triacyl-glycerides during storage, the fatty acids (C18:1 and C18:2) decreased [6,14]. This indicated that some fatty acids oxidized during storage, and this finding may be mirrored in this study by the higher fat acidity in the maize stored for 4 years. Previous studies also observed that the long-term storage of maize increased lipid oxidation and FFA [31]. A recent study confirmed the findings that the fat acidity increased, and fatty acids reduced in maize stored from 2 to 5 years [30]. The changes in fat acidity, CAT and POD activities and fatty acids may indicate that the maize was prone to oxidative rancidity during the storage and could lead to oxidative stress in broilers. The absence of difference in mycotoxin levels indicated that the storage condition might be suitable and only the duration may be the key factor for the changes in chemical composition and nutritive value. No effect of storage duration (2 to 4 years) on the content of mycotoxins in the maize was observed in a previous study [5].

### Growth performance

Oxidative stress could lead to biological damage, several pathological conditions, and depressed poultry growth [32]. The present study found that ADFI throughout the experiment was decreased in response to the stale maize diets. In agreement with our results, maize stored for 4 years lowered FCR during d 0 to 21 and 22 to 42 [30]. We failed to observe a negative effect on ADG in this study, which was inconsistent with the previous findings [30], which found that the storage duration had a significant effect on body weight gain (BWG). However, if we just compared the data obtained by previous study in maize stored for 2 and 4 years [30], respectively, no effect was observed in BWG and only the maize stored for 5 years decreased BWG in broilers. The reduced FCR in this study might mainly be due to the decreased ADFI. The fat acidity and CAT significantly correlated with broiler performance, indicating that these could be used as indexes to evaluate the quality of stored maize [30]. The mechanism underlying this difference required further investigation. Therefore, this study was further conducted to focus the effects of the stale maize diets on immunity and antioxidant capacity.

### Relative organ weight

Measurement of digestive and immune organ weights may reflect the digestive and immune status in broilers [33,34]. Such related organs include thymus, bursa of Fabricius and spleen. The spleen is also considered an essential lymphoid organ that plays an important role in cell-mediated immunity for its function in the development of suppressor T cells. Previous studies have shown that oxidative stress can cause adverse effects on the health status in poultry [35]. Feeding 100% stale maize diets reduced the relative weight of spleen, bursa of fabricius and thymus in broilers on d 21 and that of spleen on d 42 in the current study, which agreed with a previous study [5]. They found that the maize stored for 4 years decreased the relative weight of immune organs on d 21 and 42 in broilers. The decreased relative weight of immune organs may mean that the immunity of broilers is damaged due to the lipid peroxidation. These are similar to the previous results, which observed that the oxidative stress might impair the optimal functioning of the immune system [36]. Interestingly, the relative weight of liver on d 21 reduced, while the relative weight of pancreas and gizzard on d 42 increased in the present study. Although the exact reason was not clear, we supposed that the nutritive value of maize decreased during long-term storage, the broilers need compensatory stimulation of the pancreatic juice secretion to enhance digestive function and meet nutrient requirements.

### Intestinal mucosal morphology

The small intestinal villus is the main place for digestion and absorption in animals. Therefore, the maintenance of a healthy small intestine is vital for the following three functions: nutrition, immune system and gut microbiota. Villous atrophy meant that the villus absorptive cells reduced and the secretory cells increased causing a deterioration of absorptive capacity. The dietary treatment did not affect the duodenum morphology in this study on d 21 or 42, but partly influenced the jejunum and ileum morphology on d 21 or 42. Feeding 100% stale maize diets reduced VH and VH/CD in jejunum on d 21 and 42 as well as VH/CD in ileum on d 42 in the present study, which was similar to the previous findings [37]. They observed that the small intestine had decreased VH and CD in the majority of the oxidative stress cases. Oxidative stress might impair the protective barriers against pathogens entry [36]. We suppose that the lipid peroxidation in stale maize may stimulate the intestinal mucosa of broilers and lead to oxidative stress, which increased energy demand of the body as the free radicals damaged the intestinal mucosa. The VH/CD represents the digestive and absorption functions of the small intestine. The decreased VH/CD in jejunum and ileum in this study indicated that feeding stale maize diets damaged the mucous membrane of small intestine to a certain extent and decreased the absorptive capacity.

### Serum immunological parameters

The peripheral blood T lymphocyte proliferation was one of key immunity variables. Lymphocyte proliferation decreased in broilers fed stale maize diets in this study, which may have resulted from oxidative stress and greater production of reactive oxygen species. This could promote the release of glucocorticoids and inhibit immune cell proliferation. Similarly, the 100% stale maize stored for 4 years decreased the peripheral blood T lymphocyte proliferation (stimulation index by ConA) in broilers [5]. ConA is the inducer of T lymphocyte proliferation [34]. The ANAE+ percentage, which was in fact the T lymphocyte percentage and, thus, an indicator of cellular immunity, was decreased by the stale maize diets in the present study. The T lymphocyte activation and maturation was closely related to thymus reticular cells, and the decrease in the thymus T lymphocytes was the main reason for ANAE+ reduction. Therefore, the decrease in relative thymus weight in broilers fed stale maize diets may account for the reduction in ANAE+. IgG, widely occuring in the body, is involved in the immune response. Feeding 100% stale maize diets decreased serum IgG on d 21 and 42 in this study, which agreed with previous result [5]. The serum lysozyme activity is an important index of evaluating poultry nonspecific immune function and can be used as consuming system function index, which is affected by many substances, including fatty acids [38–40]. The decreased lysozyme activity on d 21 in the current study was consistent with the previous findings, which showed that the 100% stale maize stored for 4 years had lower serum lysozyme activity in broilers [5]. The avian influenza antibody H_5_N_1_ titer on d 21 was reduced in broilers fed stale maize diets in this study. To the best of our knowledge, there is no study about the effects of stale maize on Avian Influenza antibody H_5_N_1_ titer. But a similar result was observed by other researchers, who reported that the serum antibody titer of Newcastle disease of broilers decreased by the stale maize stored for 4 years [5]. The decreased IgG, ADAE+ and lymphocyte proliferation, lysozyme activity and avian influenza antibody H_5_N_1_ titer may suggest that the stale maize inhibits phagocytes activation and the activity of antibodies, eventually reduces the immunity in broilers. The mechanism of this change may be that lipid peroxidation damages the structure and function of the immune cell membrane, leading to the decline of the body’s immunity [35].

### Serum cytokines

Immune cell interaction can be mediated by cytokines, and they act as immunomodulating agents regulating both innate and adaptive immunity response to antigens and infectious agents [41]. The IL-2, which is the indispensable material in the immune response process, could stimulate the proliferation of activated natural killer cells, B lymphocytes, T lymphocytes, and antibody production. The IL-6 mainly affects the production of IgA, IgM, and IgG. The serum IL-6 was decreased by the stale maize diets, which can mirror the lower IgG in this study. The stale maize diets reduced the proinflammatory cytokines (IL-2, IFN-γ, and TNF-α), which can activate phagocytosis by macrophages, indicating that it was detrimental to the T lymphocyte proliferation and immunity. Lipid peroxidation can destroy the cellular structure and function, ultimately affecting antigen and antibody on the surface of the immune cell membrane thereby influencing the quantity and distribution of lymphatic factors, such as secretion of antibody and immune function [10,35,42].

### Serum antioxidant capacity

The SOD, GSH-PX, and T-AOC as the key enzymes of antioxidant system play a crucial role in eliminating free radicals, reducing oxidative damage and maintaining cell structure. The activities of SOD and GSH-PX and T-AOC in the serum were decreased, whereas the level of MDA increased under oxidative stress [43]. In the present study, feeding stale maize diets reduced T-AOC, SOD, and GSH-PX, whereas increased the content of MDA, which agreed with previous study [44]. They reported that with the increasing storage time, the levels of T-AOC, SOD, and GSH-PX decreased, while the level of MDA increased in broilers fed diets containing stale maize stored from 0 to 5 years. The lower T-AOC in stale maize treatment may indicate that the stale maize seriously damages the antioxidant system of broilers. The MDA is the main product of lipid peroxide degradation, which reflects the rate and intensity of lipid peroxide production and the degree of lipid peroxide as well as the degree of free radical attack in cells. The higher MDA in stale maize treatment may mean that the stale maize can lead to increased lipid peroxidation and oxidative damage in broilers. The changes in T-AOC and MDA content may be due to the decreased antioxidant enzyme activity and function in broilers, which result in the accumulation of free radicals and FFA content. The SOD and GSH-PX are the important antioxidant enzymes, whose activities can indirectly reflect the ability to remove free radicals. The stale maize diets reduced SOD and GSH-PX activities, showing that the antioxidant function of broilers was damaged. Similarly, the oxidative stress decreased SOD and GSH-PX activities in broilers [45]. This may be the main reason for the decrease in T-AOC in this study. Furthermore, the long-term storage maize increased the content of lipid peroxide and FFA, and high level of FFA was also one of the reasons for lipid peroxidation [31]. We observed a decreased CAT and POD activities and increased fat acidity in the present study, which can mirror the changes in antioxidant function. With the increasing storage time, the FFA in the maize increased, and then the free radicals accumulated, which lead to the reduced antioxidant ability [46]. The intake of the stale maize diets containing exogenous increased free radicals in broilers can increase the probability of lipid peroxidation and the number of PUFA in cell membrane, which might reduce the antioxidant capacity of the broilers and result in oxidative damage to the immune response cells [31,46–48]. The decreased immunity in this study can mirror this aspect.

## CONCLUSION

Considering the data obtained herein and the above discussion, feeding 100% stale maize diets used in this study reduced ADFI and FCR, caused detrimental effects on immunity and antioxidant capacity, and altered relative organ weight and intestinal morphology in broilers. Besides, the oxidative stress of stale maize manifested in the fat acidity and the decreased activities of CAT and POD might be the main reason for the adverse effects on the broilers. Further studies are needed to determine the effects of stale maize on meat quality in broilers.

## Figures and Tables

**Table 1:** Diet composition (as-fed basis)

Items	Starter1)	Grower1)
Ingredients (%)		
Maize	56.06	59.85
Soybean meal (CP 44%)	31.50	25.45
Corn gluten meal (CP 60%)	4.65	5.02
Tallow	3.50	5.50
Limestone	1.00	1.00
Dicalcium phosphate	2.08	1.93
Salt	0.40	0.40
L-Lys (78%)	0.20	0.20
DL-Met (99%)	0.20	0.20
L-Thr (98.5%)	0.15	0.15
Choline chloride (60%)	0.10	0.10
Vitamin premix2)	0.10	0.10
Trace mineral premix3)	0.10	0.10
Analytical composition		
ME4) (kcal/kg)	3,050	3,200
CP (%)	22.03	19.98
Lys (%)	1.17	1.05
Met (%)	0.59	0.55
Ca (%)	0.93	0.88
Total P (%)	0.72	0.67

CP, crude protein; ME, metabolizable energy.

1) Provided starter diets during d 0 to 21; provided grower diets during d 22 to 42.

2) Provided per kilogram of diet: 15,000 IU of vitamin A, 3,750 IU of vitamin D_3_, 37.5 mg of vitamin E, 2.55 mg of vitamin K_3_, 3 mg of thiamin, 7.5 mg of riboflavin, 4.5 mg of vitamin B_6_, 24 μg of vitamin B_12_, 51 mg of niacin, 1.5 mg of folic acid, 0.2 mg of biotin, and 13.5 mg of pantothenic acid.

3) Provided per kilogram of diet: 37.5 mg Zn (as ZnSO_4_); 37.5 mg Mn (as MnO_2_); 37.5 mg Fe (as FeSO_4_·7H_2_O); 3.75 mg Cu (as CuSO_4_·5H_2_O); 0.83 mg I (as KI); and 0.23 mg Se (as Na_2_SeO_3_·5H_2_O).

4) Calculated values.

**Table 2:** Nutrient content and trait of maize (as-fed basis)

Items	Control maize1)	Stale maize1)	SEM	p-value
Moisture (%)	11.74	11.02	0.27	0.65
Unit weight (g/L)	724.1	719.2	8.23	0.43
CP (%)	7.03	7.13	0.10	0.87
Starch (%)	58.88	54.74	1.05	0.03
FAV (mg KOH/100 g)	44.02	91.51	3.78	0.01
CAT activity (mg H_2_O_2_/g)	173.4	72.1	5.32	0.02
POD activity (U/g per min)	510.4	191.2	18.65	0.01
Oleic acid C18:1 (mg/g)	6.93	5.24	0.41	0.03
Linoleic acid C18:2 (mg/g)	12.82	9.78	0.67	0.04
Linolenic acid C18:3 (mg/g)	0.33	0.24	0.03	0.18
AFB_1_ (μg/kg)	3.91	4.26	0.08	0.44
OA (μg/kg)	4.02	4.65	0.06	0.61
T2 (mg/kg)	0.08	0.12	0.02	0.25
FM (mg/kg)	1.26	2.43	0.05	0.39
DON (mg/kg)	0.33	0.38	0.02	0.68
ZEA (mg/kg)	0.13	0.17	0.01	0.29

Means represent 10 samples per test maize (n = 10/group).

SEM, standard error of the means; CP, crude protein; FAV, fat acidity value; CAT, catalase; POD, peroxidase; AFB_1_, aflatoxin B_1_; OA, ochratoxin; T2, T2 toxins; FM, fumonisins; DON, deoxynivalenol; ZEA, zearalenone.

1) The control maize and stale maize stored for 1 and 4 years, respectively.

a,b Means with different superscripts in a row differ significantly (p<0.05).

**Table 3:** Effects of stale maize on growth performance in broilers

Items	CON1)	Stale maize1)	SEM	p-value
Initial BW (g)	45.4	45.5	0.02	0.38
Final BW (g)	2,607	2,578	15.1	0.19
ADG (g)				
d 0 to 21	35.4	35.6	0.19	0.34
d 22 to 42	86.7	85.0	0.52	0.12
d 0 to 42	61.0	60.3	0.33	0.43
ADFI (g)				
d 0 to 21	54.7^a^	50.2^b^	0.21	<0.01
d 22 to 42	155^a^	151^b^	0.80	0.02
d 0 to 42	105^a^	101^b^	0.42	0.02
FCR				
d 0 to 21	1.55^a^	1.41^b^	0.01	<0.01
d 22 to 42	1.79	1.78	0.01	0.26
d 0 to 42	1.72^a^	1.67^b^	0.01	0.03

Means represent 20 cages of broilers with 20 birds per cage (n = 20/group).

SEM, standard error of the means; BW, body weight; ADG, average daily gain; ADFI, average daily feed intake; FCR, feed conversion ratio.

1) CON, basal diet; Stale maize, basal diet containing 100% stale maize.

a,b Means with different superscripts in a row differ significantly (p<0.05).

**Table 4:** Effects of stale maize on relative organ weight (% of BW) in broilers

Items	CON1)	Stale maize1)	SEM	p-value
Digestive organs (%)				
Liver				
d 21	3.41^a^	3.09^b^	0.06	<0.01
d 42	2.31	2.28	0.05	0.54
Pancreas				
d 21	0.45	0.39	0.02	0.23
d 42	0.33^b^	0.37^a^	0.01	0.04
Proventriculus				
d 21	0.72	0.70	0.01	0.17
d 42	0.38	0.39	0.01	0.76
Gizzard				
d 21	3.12	3.20	0.09	0.60
d 42	2.61^b^	2.79^a^	0.06	0.04
Immune organs (%)				
Spleen				
d 21	1.08^a^	0.87^b^	0.04	0.04
d 42	1.36^a^	1.21^b^	0.04	0.04
Bursa of fabricius				
d 21	1.70^a^	1.25^b^	0.08	0.02
d 42	1.06	0.99	0.11	0.80
Thymus				
d 21	4.38^a^	4.06^b^	0.09	0.03
d 42	3.80	3.58	0.08	0.22

Means represent 20 cages of broilers with 4 birds per cage (n = 20/group).

BW, body weight; SEM, Standard error of the means.

1) CON, basal diet; Stale maize, basal diet containing 100% stale maize.

a,b Means with different superscripts in a row differ significantly (p<0.05).

**Table 5:** Effects of stale maize on intestinal mucosal morphology in broilers

Items	CON1)	Stale maize1)	SEM	p-value
Duodenum				
Villus height (μm)				
d 21	1,130	1,058	32.1	0.24
d 42	1,262	1,221	26.0	0.57
Crypt depth (μm)				
d 21	215	229	10.2	0.29
d 42	236	251	8.84	0.37
VH/CD				
d 21	5.26	4.62	0.37	0.26
d 42	5.34	4.86	0.25	0.13
Jejunum				
Villus height (μm)				
d 21	619^a^	547^b^	16.3	0.04
d 42	768^a^	678^b^	12.0	0.02
Crypt depth (μm)				
d 21	175	189	7.41	0.76
d 42	246	263	5.98	0.32
VH/CD				
d 21	3.54^a^	2.89^b^	0.28	0.04
d 42	3.12^a^	2.57^b^	0.19	0.04
Ileum				
Villus height (μm)				
d 21	548	497	18.9	0.21
d 42	602	569	15.4	0.19
Crypt depth (μm)				
d 21	148	152	7.80	0.46
d 42	123	138	5.54	0.30
VH/CD				
d 21	3.70	3.27	0.25	0.33
d 42	4.89^a^	4.12^b^	0.12	0.03

Means represent 20 cages of broilers with 4 birds per cage (n = 20/group).

SEM, standard error of the means; VH, villus height; CD, crypt depth.

1) CON, basal diet; Stale maize, basal diet containing 100% stale maize.

a,b Means with different superscripts in a row differ significantly (p<0.05).

**Table 6:** Effects of stale maize on the serum immunity in broilers

Items	CON1)	Stale maize1)	SEM	p-value
IgG (μg/mL)				
d 21	107^a^	80.0^b^	4.12	0.02
d 42	113^a^	86.2^b^	4.03	0.03
Lysozyme activity (μg/mL)				
d 21	2.28^a^	1.42^b^	0.08	<0.01
d 42	1.48	1.19	0.10	0.17
ANAE^+^ (%)				
d 21	44.3^a^	35.7^b^	1.88	0.02
d 42	58.3^a^	45.2^b^	2.41	0.03
Lymphocyte proliferation (%)				
d 21	43.3^a^	35.2^b^	2.28	0.04
d 42	45.4^a^	37.7^b^	2.02	0.03
Avian Influenza antibody H_5_N_1_ titer, log_2_				
d 21	2.18^a^	1.39^b^	0.16	0.03
d 42	2.15	2.08	0.14	0.88

Means represent 20 cages of broilers with 4 birds per cage (n = 20/group).

SEM, standard error of the means; IgG, immunoglobin G; ANAE^+^, acid α-naphthylacetate esterase positive ratios.

1) CON, basal diet; Stale maize, basal diet containing 100% stale maize.

a,b Means with different superscripts in a row differ significantly (p<0.05).

**Table 7:** Effects of stale maize on the serum cytokines in broilers

Items	CON1)	Stale maize1)	SEM	p-value
IL-2 (g/mL)				
d 21	104^a^	91.4^b^	3.31	0.03
d 42	133	127	3.85	0.32
IL-6 (ng/mL)				
d 21	16.8^a^	14.3^b^	0.31	<0.01
d 42	20.8^a^	19.7^b^	0.28	0.04
IFN-γ (ng/mL)				
d 21	18.2^a^	16.1^b^	0.20	0.02
d 42	18.5	18.3	0.22	0.76
TNF-α (pg/mL)				
d 21	40.9^a^	38.4^b^	0.65	0.04
d 42	21.8	20.7	0.71	0.18

Means represent 20 cages of broilers with 4 birds per cage (n = 20/group).

1) CON, basal diet; Stale maize, basal diet containing 100% stale maize.

SEM, standard error of the means; IL, interleukin; IFN-γ, interferon-γ; TNF-α, tumor necrosis factor-α.

a,b Means with different superscripts in a row differ significantly (p<0.05).

**Table 8:** Effects of stale maize on the serum antioxidant capacity in broilers

Items	CON1)	Stale maize1)	SEM	p-value
T-AOC (U/mL)				
d 21	15.4	14.2	2.31	0.41
d 42	21.0^a^	12.2^b^	2.78	0.04
SOD (U/mL)				
d 21	176a	157^b^	5.48	0.04
d 42	169^a^	141^b^	5.20	0.03
GSH-PX (U/mL)				
d 21	292^a^	270^b^	7.02	0.04
d 42	304^a^	268^b^	6.53	0.02
MDA (nmol/mL)				
d 21	2.98^a^	3.86^b^	0.32	0.04
d 42	3.04^a^	4.80^b^	0.27	<0.01

Means represent 20 cages of broilers with 4 birds per cage (n = 20/group).

SEM, standard error of the means; T-AOC, total antioxidative capacity; SOD, superoxide dismutase; GSH-PX, glutathione peroxidase; MDA, malondialdehyde.

1) CON, basal diet; Stale maize, basal diet containing 100% stale maize.

a,b Means with different superscripts in a row differ significantly (p<0.05).
